# EAT‐Lancet Diet Adherence in Patients With Type 2 Diabetes: A Cross‐Sectional Study

**DOI:** 10.1002/fsn3.70650

**Published:** 2025-07-18

**Authors:** Meltem Karabicak, Aysun Yuksel

**Affiliations:** ^1^ Sisli Hamidiye Etfal Training and Research Hospital Istanbul Türkiye; ^2^ Department of Nutrition and Dietetics Istanbul Medeniyet University Istanbul Türkiye

**Keywords:** EAT‐Lancet diet, planetary diet, sustainability, sustainable nutrition, type 2 diabetes

## Abstract

The aim of this cross‐sectional study was to determine the adherence of individuals with type 2 diabetes to the EAT‐Lancet diet and examine the relationship between dietary adherence and various health parameters. The study was conducted with 385 individuals with type 2 diabetes at a training and research hospital between August and November 2023. Data on general information, biochemical parameters, anthropometric measurements, 24‐h dietary recall, and food frequency questionnaire were collected. The EAT‐Lancet Diet Index was used to classify dietary adherence. Of the participants, 52.7% were female, and the mean age was 54.42 (SD 11.53) years. Higher adherence to the EAT‐Lancet diet was associated with higher dietary fiber intake and lower total energy, protein, and fat intake. A statistically significant positive correlation was found between the EAT‐Lancet Diet Index and diabetes duration (*p* = 0.03), while a negative correlation was found between the index and fasting blood glucose levels (*p* = 0.02). Participants with higher adherence also had better glycemic control and lower HbA1c levels. Sociodemographic factors such as age influenced dietary adherence, with older individuals showing higher adherence. It is concluded that adherence to the EAT‐Lancet diet is significantly associated with improved glycemic control and better health outcomes in individuals with type 2 diabetes. Promoting this sustainable diet through policy interventions and educational programs can enhance public health and environmental sustainability. Incorporating sustainable nutrition recommendations into dietary guidelines is recommended to improve adherence among diabetic patients.

## Graphical abstract

In a sample of 385 adults with type 2 diabetes, higher EAT‐Lancet Diet Index scores correlated with increased dietary fiber intake and reduced total energy, protein, and fat consumption. Improved adherence was also associated with significantly lower fasting blood glucose and HbA1c levels. These results suggest that promoting a sustainable, plant‐forward dietary pattern may enhance glycemic control and overall metabolic health in this population.

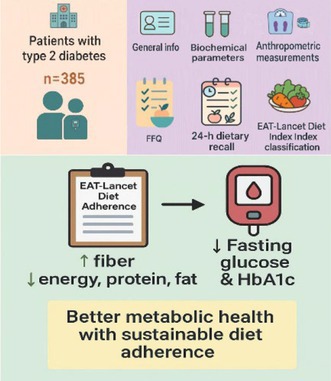

**Abbreviations**
BeBIS: nutrition information system
BMI: body mass index
HbA1c: glycosylated hemoglobin

## 1 Introduction

Type 2 diabetes is a heterogeneous chronic disease with a complex pathogenesis, characterized by high blood glucose levels or hyperglycemia. Hyperglycemia causes metabolic dysfunctions and leads to diabetic complications (Banday et al. 2020). Over the past 30 years, the prevalence of type 2 diabetes has increased globally, reaching epidemic levels. This has resulted in a global crisis threatening human health, public health systems, and the world economy. Currently, it is estimated that approximately one in every 11 adults (537 million) worldwide has diabetes, with projections indicating an increase to 643 million by 2030 (Ogurtsova et al. 2022; Zheng et al. 2018). The prevalence of diabetes is rising in Türkiye as well (Satman et al. 2023).

These increases are largely attributed to lifestyle changes, including higher consumption of red and processed meats, as well as low‐quality carbohydrates such as sugary drinks and refined grains, and dietary patterns lacking adequate plant nutrients (Tinajero and Malik 2021). Management of diabetes involves lifestyle interventions such as dietary changes and physical activity, alongside pharmaceutical treatments. The basis of lifestyle changes is a balanced diet and an appropriate diet model (ElSayed et al. 2023a, 2023b).

There are many dietary models long used in the treatment of diabetes. Among these, the EAT‐Lancet diet, designed in 2019, has attracted attention due to its potential contributions to a sustainable environment, public health, and nutrition (Lin et al. 2023). The EAT‐Lancet diet, also known as the planetary health diet, was developed by the EAT‐Lancet commission as a universal reference diet (Willett et al. 2019). The aim is to improve human health while optimizing environmental sustainability, characterized by high consumption of plant‐based foods; limited intake of animal products, refined foods, added sugars, and refined grains; consumption of healthy fats; adequate nutritional intake; flexibility; and environmental sustainability (Willett et al. 2019).

Adherence to the EAT‐Lancet diet has the potential to prevent non‐communicable chronic diseases such as diabetes, as well as reduce the environmental impacts of food production (Willett et al. 2019). This is particularly important for ensuring healthy nutrition and sustainable development for the world's population, projected to reach 10 billion by 2050 (Grosso et al. 2020; Pérez‐Escamilla 2017). Studies have been performed to investigate the health effects of the EAT‐Lancet diet on various conditions, including metabolic health, cardiovascular diseases, stroke, cancer, cognitive function and performance, metabolic dysfunction–associated steatotic liver disease, inflammatory bowel disease, and diabetes risk (Cacau et al. 2024; Ibsen et al. 2022; Kuipers 2025; Lin et al. 2023; Tavares et al. 2023; Ye et al. 2025). The diet's benefits on diabetes are associated with high consumption of fruits, vegetables, whole grains, legumes, unsaturated oils, and nuts, and low consumption of meat and added sugars. The plant‐based, high‐fiber EAT‐Lancet diet supports weight control, lower obesity rates, glycemic control, and insulin sensitivity, which are critical in diabetes management (Lin et al. 2023; Mambrini et al. 2025; Ojo et al. 2023). The components of this diet also have a favorable effect on blood sugar levels (Muszalska et al. 2025).

This diet may not have the same effect in every country and may have different effects based on differences in countries' dietary culture and income levels (Aydogdu and Gezmen Karadag 2025). However, adherence to this diet among diabetic patients in Türkiye has not been previously determined.

Most studies on diet and health effects have been conducted in populations of high socioeconomic status, highlighting the need for research in diverse populations. The purpose of the present study was to evaluate adherence among patients with type 2 diabetes to the EAT‐Lancet diet and to examine the relationship between this adherence and various demographic, anthropometric, and biochemical parameters.

## 2 Methods

### 2.1 Ethics Statement

This study was conducted in accordance with the guidelines laid down in the Declaration of Helsinki, and all procedures involving human subjects were approved by the Hamidiye Scientific Research Ethics Committee (meeting number 2023/11, decision number 11/3 on 26.05.2023), University of Health Sciences, Istanbul, Türkiye. Written informed consent was obtained from all participants.

### 2.2 Study Design

This cross‐sectional study was conducted between August and November 2023 at the nutrition outpatient clinic of a training and research hospital.

### 2.3 Participants

The sample size of the study was calculated with a 5% margin of error and a 95% confidence interval in the unknown population. Accordingly, it was appropriate to include at least 385 patients. The study included 406 voluntary participants over the age of 18 who were literate and diagnosed with type 2 diabetes. These individuals were referred to the nutrition outpatient clinic of a training and research hospital, a Ministry of Health hospital in Istanbul by their physicians. The exclusion criteria included being pregnant and lactating.

### 2.4 Data Collection

A questionnaire form consisting of 4 sections was applied by face‐to‐face interview. During the interview, demographic and health information, dietary habits, 24‐h dietary recall, and food consumption frequencies were obtained. In addition, fasting blood glucose (mg/dL) and HbA1c levels (%) were recorded, and anthropometric measurements were made.

### 2.5 Dietary Assessment

In this study, 24‐h dietary recall and food frequency questionnaire were used to assess dietary status. One‐day 24‐h recall interviews were conducted to obtain detailed food intake information. Individuals were first asked what they consume. Then the amounts of what they consumed were recorded (Thompson and Subar 2017). The “Food and Nutrition Photo Catalog” was used to determine the size and quantity of the foods consumed by individuals (Rakicioglu et al. 2012). The food frequency questionnaire adapted to Türkiye was developed in accordance with the dietary habits of the Turkish population and a validity and reliability study was performed (Gunes et al. 2015; Pekcan 2008). The frequency of consumption of some nutrients included in the EAT‐Lancet diet was also questioned (Stubbendorff et al. 2022). 24‐h dietary recall and food frequency data were analyzed and averaged using the Nutrition Information System (BeBIS) program. (The results have been presented as a File S1). Adherence to the EAT‐Lancet diet was assessed using the EAT‐Lancet Diet Index developed by Stubbendorff et al. (2022).

### 2.6 Biochemical and Anthropometric Assessments

Fasting blood glucose (mg/dL) and HbA1c levels (%) were recorded from the patient information system of the hospital where the study was conducted.

During anthropometric measurements, the dietitian with the TANITA measured body weight (kg). Height (cm) was measured with a stadiometer with the feet side by side and the head parallel to the ground (Gibson 2005). Body Mass Index (kg/m^2^) was calculated by dividing body weight by the square of height in meters. Individuals were classified as underweight, normal, overweight, or obese according to the World Health Organization classification (World Health Organization 2010). The dietitian took waist and hip measurements using a non‐flexible tape measure while the individual was standing with arms at both sides and feet at the side Waist circumference was measured from the center of the cristiliac and lower ribs (World Health Organization 2011). In addition, hip circumference was taken with the tape measure passing through the highest point on the hip. Waist/hip ratio was calculated as waist circumference in cm divided by hip circumference (Gibson 2005; World Health Organization 2011).

### 2.7 EAT‐Lancet Diet Index

This index is used to measure adherence to the target intake and reference ranges recommended by the EAT‐Lancet commission for different nutrients in the EAT‐Lancet diet (Willett et al. 2019). The EAT‐Lancet Diet Index includes 14 food groups, including 7 emphasized nutrients and 7 limited nutrients. Whole grains, vegetables, fruits, legumes, nuts, fish, and unsaturated oils are emphasized, and increased intake increases dietary adherence, while potatoes, beef and lamb, poultry, pork, eggs, dairy products, and added sugar are limited, and increased intake increases dietary non‐adherence. Based on intake, each food group in the index is scored from 0 points (low adherence) to 3 points (high adherence). Dietary adherence is evaluated according to total scores ranging from 0 (non‐adherence) to 42 (highest adherence) obtained from the 14 food groups. As the score increases, dietary adherence increases, and as the score decreases, dietary adherence decreases.

The scoring of the 14 food groups in the EAT‐Lancet Diet Index is given below.

#### 2.7.1 Emphasized Intakes

– Vegetables: In the EAT‐Lancet diet, the daily reference intake of vegetables is 200–600 g and the target intake is 300 g. According to the Diet Index, 3 points are given for daily intake of more than 300 g of vegetables, 2 points for 200–300 g, 1 point for 100–200 g, and 0 points for less than 100 g.
– Fruits: The target intake of fruits with a reference intake of 100–300 g per day in the diet is 200 g per day. When scoring, 3 points are given if the daily fruit intake is more than 200 g, 2 points if it is 100–200 g, 1 point if it is 50–100 g, and 0 points if it is less than 50 g.
– Unsaturated oils: The target intake of unsaturated oils in the diet is 40 g per day, with a reference intake of 20–80 g per day. Three points are given for daily intake of more than 40 g of unsaturated oils, 2 points for 20–40 g, 1 point for 10–20 g, and 0 points for less than 10 g.
– Legumes: The recommended daily intake of legumes is 0–150 g and the target intake is 75 g. In scoring, 3 points are given for legume intake of more than 75 g, 2 points for 37.5–75 g, 1 point for 18.75–37.5 g, and 0 points for less than 18.75 g.
– Nuts: In the planetary diet, the reference intake is 0–100 g per day and the target intake is 50 g per day. Three points are given for an intake of more than 50 g of nuts per day, 2 points for 25–50 g, 1 point for 12.5–25 g, and 0 points for less than 12.5 g.
– Whole grains: The target whole grain intake in the EAT‐Lancet diet is 232 g/day. Whole grains are scored as 3 points if the daily intake is above 232 g, 2 points if it is between 116 and 232 g, 1 point if it is between 58 and 116 g, and 0 points if it is below 58 g.
– Fish: Reference intake is 0–100 g per day and target intake is 28 g per day. Daily intake of more than 28 g is scored 3 points, 14–28 g is scored 2 points, 7–14 g is scored 1 point, and less than 7 g is scored 0 points.

#### 2.7.2 Limited Intakes

– Beef and lamb: The reference intake of beef and lamb, which are foods limited in the diet, is 0–14 g per day, while the target intake is 7 g. When scoring, 3 points are given for a daily meat intake of less than 7 g, 2 points for 7–14 g, 1 point for 14–28 g, and 0 points for more than 28 g.
– Pork: The reference intake of pork in the EAT‐Lancet diet is 0–14 g per day, while the target intake is 7 g per day. When scoring, 3 points are given if the daily pork intake is less than 7 g, 2 points in the range of 7–14 g, 1 point in the range of 14–28 g, and 0 points if it is more than 28 g.
– Poultry: The recommended dietary intake of poultry is 0–58 g and the target intake is 29 g. Three points are given for poultry intake less than 29 g, 2 points for 29–58 g, 1 point for 58–116 g, and 0 points for more than 116 g.
– Eggs: According to the EAT‐Lancet diet, the daily reference intake of eggs is 0–25 g per day and the target intake is 13 g per day. When scoring, 3 points are given if the egg intake is less than 13 g per day, 2 points if it is between 13 and 25 g, 1 point if it is 25–50 g, and 0 points if it is over 50 g.
– Dairy: The reference intake of dairy in the EAT‐Lancet diet is 0–500 g per day and the target intake is 250 g per day. When calculating, 3 points are given for an intake of less than 250 g of dairy per day, 2 points for 250–500 g, 1 point for 500–1000 g, and 0 points for more than 1000 g.
– Potatoes: The reference range is 0–100 g per day and the target intake is 50 g per day. If the daily intake of potatoes is below 50 g, 3 points are given; if it is between 50 and 100 g, 2 points are given; if it is between 100 and 200 g, 1 point is given; and if it is above 200 g, 0 points are given.
– Added sugar: The daily reference amount of added sugar in the EAT‐Lancet diet is 0–31 g and the target amount is 31 g. When scoring, 3 points are given if the amount of sugar intake is less than 31 g, 2 points if it is between 31 and 62 g, 1 point if it is between 62 and 124 g, and 0 points if it is over 124 g.

### 2.8 Classification

The EAT‐Lancet Diet Index was used to determine dietary adherence, and the participants were classified. The classification was based on the objectives of making the groups as similar as possible in size while maintaining the same range of scores and avoiding groups that were too small to prevent unreliable dietary data. The participants were classified according to their EAT‐Lancet Diet Index scores: ≤ 13, 14–16, 17–19, 20–22, and ≥ 23 points, with higher scores indicating greater adherence to the diet.

### 2.9 Statistical Analysis

Before the statistical analyses, frequency analysis was performed to assess the accuracy of data entry. At this stage, no incorrect or incomplete items were detected in the data. Then coding/reverse coding was performed for the EAT‐Lancet Diet Index, and the total score for the index was calculated. The EAT‐Lancet Diet Index scores were then converted into standard z scores, and, as a result of the conversion, no individual outside the ±3 range was identified. In total, data from 385 individuals were collected, and all of these data were used for analysis.

All statistical analyses were performed using IBM SPSS version 25.0 (SPSS Inc., Chicago, IL, USA). Continuous variables are presented as mean and standard deviation (SD), while categorical variables are presented as numbers (*n*) and percentages (%). Normality of the EAT‐Lancet Diet Index scores was confirmed by skewness and kurtosis values and histogram analysis. Pearson's correlation analysis was used to assess relationships between the EAT‐Lancet Diet Index scores and numerical variables. In order to determine whether there was a significant difference between EAT‐Lancet Diet Index total scores and demographic variables of individuals, the independent samples t test was applied for variables with two groups, while one‐way ANOVA was applied for variables with 3 or more groups. Additionally, to control for potential confounding variables, a multiple linear regression analysis was conducted with EAT‐Lancet Diet Index score as the dependent variable and age, duration of diabetes, body mass index (BMI), fasting blood glucose, and HbA1c as independent variables. The chi‐squared test was applied for categorical variables. Statistical significance was set at *p* < 0.05.

## 3 Results

### 3.1 Characteristics of Individuals With Type 2 Diabetes

The study included 406 individuals, and 21 individuals provided missing data. The response rate was 94.8%. Of the 385 individuals with type 2 diabetes who participated in the study, 203 were female (52.7%) and 182 were male (47.2%). The mean age of the participants was 54.42 (SD 11.53) years. The mean duration since diagnosis was 6.78 (SD 7.8) years. Moreover, 69.9% were literate or had a primary or secondary school education.

Most (61.6%) did not smoke and 90.6% did not drink alcohol. To the question, “Have you heard of the concept of sustainable nutrition?” 87.5% of the participants answered “no”. Additionally, 34.5% of the participants regularly performed physical activity. The mean body mass index (BMI) was 32.29 (SD 6.65) kg/m^2^, with 0.5% underweight, 11.9% normal, 27.0% overweight and 60.5% obese. The mean waist circumference was 110.01 (SD 14.37) cm and the mean waist/hip ratio was 0.97 (SD 0.07). The mean fasting blood glucose level was 184.41 (SD 86.49) mg/dL and the mean HbA1c level was 8.63% (SD 2.47). The mean daily energy intake was 1429.61 (SD 417.29) kcal. The average energy obtained from protein, carbohydrate, and fat was 17.23% (SD 3.06), 42.79% (SD 9.13), and 39.87% (SD 7.9), respectively. It was also established that the average daily dietary fiber intake was 16.79 (SD 6.41) g (Table 1).

**TABLE 1:** Demographic and health characteristics of individuals with type 2 diabetes^a^.

Characteristics	Individuals with type 2 diabetes (*n* = 385)	
	Mean or *n*	SD or %
Gender (%)		
Female	203	52.7
Male	182	47.2
Age (years)	54.42	11.53
Age with diabetes (years)	6.78	7.8
Education status (%)		
Literate, primary, secondary school	269	69.9
High school	71	18.4
Bachelor's degree, postgraduate	45	11.7
Smoking status (%)		
Drinking	113	29.4
Not drinking	237	61.6
Used to drink	35	9.1
Alcohol consumption status (%)		
Consume	36	9.4
Does not consume	349	90.6
Hearing of sustainable nutrition (%)		
Yes	48	12.5
No	337	87.5
Status of regular physical activity (%)		
Yes	133	34.5
No	252	65.5
BMI (kg/m^2^)	32.29	6.65
BMI classification (%)		
Underweight	2	0.5
Normal	46	11.9
Overweight	104	27
Obese	233	60.5
Waist circumference (cm)	110.01	14.37
Waist/hip ratio	0.97	0.07
Fasting blood glucose (mg/dL)	184.41	86.49
HbA1c (% level)	8.63	2.47
Energy intake (kcal)	1429.61	417.29
Carbohydrates (Energy %)	42.79	9.13
Protein (Energy %)	17.23	3.06
Fat (Energy %)	39.87	7.9
Dietary fiber intake (g)	16.79	6.41

Abbreviations: BMI, body mass index; HbA1c, glycosylated hemoglobin.

^a^ Values are presented as means and standard deviations for continuous variables, and numbers and percentages for categorical variables.

### 3.2 Consumption of Food Groups and Scoring According to the EAT‐Lancet Diet Index

The mean daily intakes (g) for the 14 food groups included in the EAT‐Lancet diet and the mean points obtained from the EAT‐Lancet Diet Index are given in Table 2.

**TABLE 2:** Mean intake amounts of food groups and mean EAT‐Lancet Diet Index points of type 2 diabetics.

Food groups	Target intakes in EAT‐Lancet diet (possible range)	Mean daily intakes (g)		Mean EAT‐Lancet Diet Index points (0–3 points)^a^	
	(g/day)	Mean	SD	Mean	SD
Whole grains	232	53.23	78.63	0.56	0.85
Potatoes	50 (0–100)	17.14	29.80	2.87	0.42
Vegetables	300 (200–600)	159.27	75.59	1.11	0.75
Fruits	200 (100–300)	118.61	100.26	1.41	1.11
Dairy	250 (0–500)	250.29	132.57	2.49	0.60
Beef and lamb	7 (0–14)	32.55	27.51	1.00	1.12
Pork	7 (0–14)	0.00	0.00	3.00	0.00
Poultry	29 (0–58)	29.71	34.10	2.51	0.85
Eggs	13 (0–25)	42.61	34.22	1.23	1.17
Fish	28 (0–100)	6.77	20.15	0.36	0.84
Legumes	75 (0–150)	18.62	24.84	0.49	0.87
Nuts	50 (0–100)	6.27	10.71	0.24	0.60
Unsaturated oils	40 (20–80)	34.85	15.03	2.19	0.73
Added sugar	31 (0–31)	33.00	45.46	2.44	0.86

^a^ Each food group is scored from 0 points (low adherence) to 3 points (high adherence) in the EAT‐Lancet Diet Index.

**FIGURE 1:**
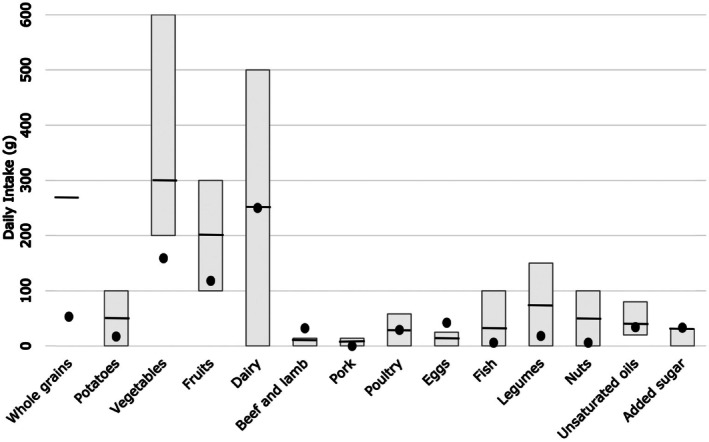
Recommendations and target intakes in the EAT‐lancet diet and mean intakes in this study.

The target and intake range for the food groups recommended in the EAT‐Lancet diet and the mean intakes from our study are shown in Figure 1.

**FIGURE 2:**
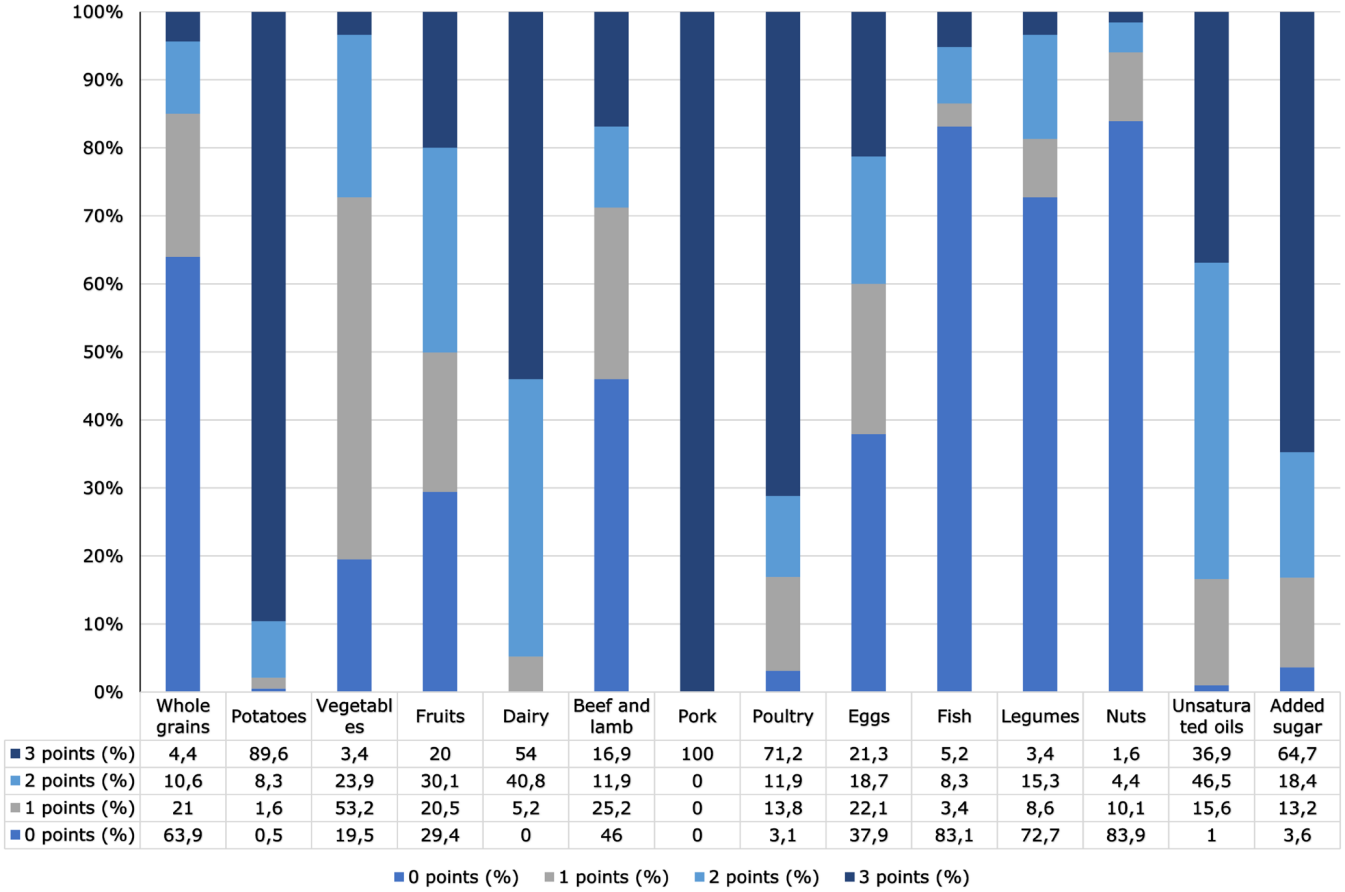
Distribution of EAT‐lancet diet index points for food groups.

Analysis of the EAT‐Lancet Diet Index scores revealed that less than 5% of the study population reached the dietary target intakes for whole grains, vegetables, legumes, and nuts. The participants reported never consuming pork. The highest adherence to the EAT‐Lancet diet was observed for pork (100%), potatoes (89.6%), poultry (71.2%), added sugar (64.7%), and dairy (54%) (Figure 2). The foods with the lowest dietary adherence were nuts (1.6%), legumes (3.4%), vegetables (3.4%), whole grains (4.4%), and fish (5.2%). The adherence order from highest to lowest was pork, potatoes, poultry, added sugar, dairy, unsaturated oils, eggs, fruits, beef and lamb, fish, whole grains, vegetables, legumes, and nuts (Figure 2).

While men were more compliant with the whole grain recommendations than women, women were observed to be more compliant with the beef, lamb, and added sugar recommendations in the EAT‐Lancet diet.

### 3.3 Classification of Individuals With Type 2 Diabetes According to Their EAT‐Lancet Diet Index Score and Comparison of Their Characteristics According to Their Adherence

The mean EAT‐Lancet Diet Index score among the participants was 21.89 (SD 3.51), ranging from 14 to 31. Women had a mean index score of 22.14 (SD 3.31), slightly higher than that of men, who had a mean score of 21.61 (SD 2.94). The analysis showed no significant difference between the total dietary index score and sex (*t* = 1.659, *p* = 0.09).

The participants were classified into four groups based on their EAT‐Lancet Diet Index scores: 3.6% (*n* = 14) scored 14–16 points, 17.9% (*n* = 69) scored 17–19 points, 37.1% (*n* = 143) scored 20–22 points, and 41.3% (*n* = 159) scored ≥ 23 points. The majority of the study population (41.3%) was found to be in the highest dietary adherence class.

In Table 3, the general characteristics, anthropometric measurements, biochemical parameters, and energy and macronutrient intake were compared according to EAT‐Lancet Diet Index scores. Type 2 diabetic participants with a score between 14 and 16 (lowest dietary adherence) had higher protein (Energy %) values compared to the others (*F* = 5.301, *p* = 0.001). Those with a dietary index score of ≥ 23 points (highest adherence) had higher dietary fiber intake (g) (*F* = 10.349, *p* < 0.001) and alcohol abstinence (*p* = 0.02) compared to the others.

**TABLE 3:** Comparison of characteristics and EAT‐Lancet Diet Index classification in type 2 diabetes^d^.

Characteristics	14–16 points (*n* = 14)		17–19 points (*n* = 69)		20–22 points (*n* = 143)		≥ 23 points (*n* = 159)		*p*
	Mean or *n*	SD or %	Mean or *n*	SD or %	Mean or *n*	SD or %	Mean or *n*	SD or %	
Age (years)	50.07	16.74	55.14	11.28	53.13	11.59	55.64	10.93	0.11^a^
Age with diabetes (years)	7.86	7.49	5.49	7.20	6.53	8.34	7.48	7.56	0.31^a^
Gender (%)									
Female	10	71.4	30	43.5	73	51	90	56.6	0.14^b^
Male	4	28.6	39	56.5	70	49	69	43.4	
Education status (%)									
Literate, primary, secondary school	5	35.6	48	69.6	104	72.8	112	70.5	0.09^b^
High school	7	50	13	18.8	22	15.4	29	18.2	
Bachelor's degree, postgraduate	2	14.3	8	11.6	17	11.9	18	11.4	
Smoking status (%)									
Drinking	4	28.6	26	37.7	39	27.3	44	27.7	0.71^b^
Not drinking	9	64.3	39	56.5	91	63.6	98	61.6	
Used to drink	1	7.1	4	5.8	13	9.1	17	10.7	
Alcohol consumption status (%)									
Consume	2	14.3	13	18.8	10	7	11	6.9	0.02^**b**^
Does not consume	12	85.7	56	81.2	133	93	148	93.1	
Hearing of sustainable nutrition (%)									
Yes	2	14.3	12	17.4	20	14	14	8.8	0.28^b^
No	12	85.7	57	82.6	123	86	145	91.2	
Status of regular physical activity (%)									
Yes	4	28.6	24	34.8	42	29.4	63	39.6	0.29^b^
No	10	71.4	45	65.2	101	70.6	96	60.4	
BMI (kg/m^2^)	32.60	5.89	32.54	6.88	31.68	6.26	32.70	6.98	0.58^a^
BMI classification (%)									
Underweight	0	0	0	0	1	0.7	1	0.6	0.81^c^
Normal	1	7.1	7	10.1	23	16.1	15	9.4	
Overweight	4	28.6	21	30.4	34	23.8	45	28.3	
Obese	9	64.3	41	59.4	85	59.4	98	61.6	
Waist circumference (cm)	108.14	12.49	110.18	13.89	108.79	15.73	111.20	13.45	0.49^a^
Waist/hip ratio	0.97	0.05	0.97	0.07	0.97	0.08	0.97	0.07	0.95^a^
Fasting blood glucose (mg/dL)	178.21	73.82	192.95	95.63	192.19	84.87	174.25	84.43	0.25^a^
HbA1c (% level)	7.96	2.57	8.81	2.51	8.88	2.50	8.37	2.40	0.21^a^
Energy intake (kcal)	1490.65	398.68	1501.99	441.05	1461.36	415.91	1364.26	403.88	0.06^a^
Carbohydrate (Energy %)	37.78	9.85	42.84	9.59	42.97	9.08	43.06	8.86	0.22^a^
Protein (Energy %)	19.07	4.11	18.17	3.17	17.13	2.96	16.76	2.88	0.001^**a**^
Fat (Energy %)	43.17	7.74	38.50	8.09	39.87	7.91	40.16	7.78	0.19^a^
Dietary fiber (g)	10.74	3.82	14.99	5.93	16.40	5.94	18.44	6.65	< 0.001^a^

Abbreviations: BMI, body mass index; HbA1c, glycosylated hemoglobin.

^a^ One Way ANOVA.

^b^ Pearson Chi‐Square.

^c^ Fisher's Exact test, *p* < 0.05.

^d^ Values are presented as mean and standard deviation for continuous variables, and number and percentage for categorical variables.

Those with high adherence to the EAT‐Lancet diet (higher score) had lower total energy intake and a diet containing a lower percentage of energy from fat, a higher percentage of energy from carbohydrates, more dietary fiber, and a lower percentage of energy from protein compared to those with low adherence (Table 3). The participants with high adherence were slightly older, less likely to smoke, and very unlikely to consume large amounts of alcohol.

The relationship between the EAT‐Lancet Dietary Index score and energy consumption and nutrients is shown in Table 4. There was a statistically significant negative correlation between EAT‐Lancet Diet Index score and protein intake (g) (*r* = −0.195, *p* < 0.001), protein (Energy%) values (*r* = −0.178, *p* < 0.001), total energy intake (kcal) (*r* = −0.130, *p* = 0.01) and fat intake (g) (*r* = −0.112, *p* = 0.02). In addition, there was a statistically significant positive correlation between EAT‐Lancet Diet Index score and dietary fiber intake (g) (*r* = 0.299, *p* < 0.001).

**TABLE 4:** Association between EAT‐Lancet diet index points and nutrients.

Energy and nutrients	EAT‐Lancet Diet Index points	
	*r*	*p*
Energy (kcal)	−0.130	0.01
Carbohydrate (g)	−0.070	0.17
Carbohydrate (Energy %)	0.064	0.21
Protein (g)	−0.195	< 0.001
Protein (Energy %)	−0.178	< 0.001
Fat (g)	−0.112	0.02
Fat (Energy %)	0.010	0.85
Dietary fiber (g)	0.299	< 0.001

*Note:* Pearson correlation test, *p* < 0.05.

According to the results of Pearson's correlation analysis, there was a statistically significant positive correlation between EAT‐Lancet Diet Index score and the duration of diabetes (*r* = 0.110, *p* = 0.03) and a negative correlation between EAT‐Lancet Diet Index score and fasting blood glucose level (mg/dL) (*r* = −0.114, *p* = 0.02) (Table 5).

**TABLE 5:** Correlation of some variables with EAT‐Lancet diet index points.

Variables	EAT‐Lancet Diet Index points	
	*r*	*p*
Age (years)	0.074	0.14
Duration of diabetes (years)	0.110	0.03
BMI (kg/m^2^)	0.007	0.89
Waist circumference (cm)	0.031	0.54
Waist/hip ratio	−0.030	0.55
Fasting blood glucose (mg/dL)	−0.114	0.02
HbA1c level (%)	−0.080	0.13

*Note:* Pearson correlation test, *p* < 0.05.

Abbreviation: BMI, body mass index.

Analyses also showed that there were positive correlations between dietary adherence (EAT‐Lancet Diet Index score) and age, BMI, and waist circumference, and negative correlations between dietary adherence and waist/hip ratio and HbA1c level (%) (Table 5).

To evaluate whether clinical and demographic factors influence adherence to the EAT‐Lancet dietary pattern, we implemented a multiple linear regression model incorporating age, diabetes duration, BMI, fasting glucose, and HbA1c. As shown in Table 6, the model did not reach statistical significance (*F*(5,367) = 2.01, *p* = 0.076), accounting for approximately 3% of the variance in the dietary index scores (*R* ^2^ = 0.03). None of the included predictors demonstrated a statistically significant association with diet adherence. However, diabetes duration exhibited a marginal trend (β = 0.10, *p* = 0.093), indicating a possible association that warrants further investigation. Model diagnostics confirmed the validity of the regression assumptions, including the absence of multicollinearity (all VIFs < 3) (Table 6).

**TABLE 6:** Predicting EAT‐Lancet Diet Score.

Predictor	*B*	SE	95% CI	β	*t*	*p*
(Intercept)	21.87	1.47	[18.98, 24.76]	0.00	14.87	< 0.001
Age (years)	0.007	0.02	[−0.02, 0.04]	0.03	0.46	0.644
Diabetes duration (years)	0.04	0.02	[−0.006, 0.08]	0.10	1.68	0.093
BMI (kg/m^2^)	−0.001	0.03	[−0.05, 0.05]	−0.002	−0.04	0.968
Fasting blood glucose (mg/dL)	−0.005	0.003	[−0.01, 0.001]	−0.13	−1.60	0.111
HbA1c (%)	0.03	0.11	[−0.18, 0.24]	0.02	0.29	0.772

*Note:* Multiple linear regression test. Results: *F*(5,367) = 2.01, *p* = 0.076, *R* ^2^ = 0.03 All assumptions met; no multicollinearity (VIFs < 3).

## 4 Discussion

The participants in a Swedish cohort study obtained a mean score of 17.9 (SD 3.4) on the EAT‐Lancet Diet Index (Stubbendorff et al. 2022). All type 2 diabetics in the present study received a mean score of 21.89 (SD 3.51) on the EAT‐Lancet Diet Index. One possible reason for the higher scores in our study compared to the Swedish study may be that the participants in our study obtained full points (3 points) on the EAT‐Lancet Diet Index for not consuming pork.

In a UK cohort study in which adherence to the EAT‐Lancet diet was examined in a population that contained a large proportion of vegetarians, it was reported that between 96.3% and 100% of the participants adhered to the recommendations for poultry, eggs, legumes, unsaturated oils, and fish (Knuppel et al. 2019). Stubbendorff et al. (2022) found that dietary adherence was highest for poultry (77% of the population) and fish (66% of the population) in a Swedish cohort. In the present study, the order from the highest to the lowest percentage of adherence to the EAT‐Lancet diet was as follows: pork (100%), potatoes (89.6%), poultry (71.2%), added sugar (64.7%), dairy (54%), unsaturated oils (36.9%), eggs (21.3%), fruits (20%), beef and lamb (16.9%), fish (5.2%), whole grains (4.4%), vegetables (3.4%), legumes (3.4%), and nuts (1.6%). While 66% of the fish consumption recommendation is met in Sweden, 5.2% is met in Türkiye, which can be attributed to the countries' dietary habits and sociocultural differences. Daily pork intake was 0 g in the whole population, and all participants scored full points on the index. This may be explained by the religious and sociocultural structure of Türkiye, where pork consumption is generally avoided.

The consumption of whole grains in the diet is associated with health outcomes such as reduced risk of type 2 diabetes by increasing nutrient and fiber intake and has been accepted as a public health recommendation (Aune et al. 2013; Xu et al. 2021; Zong et al. 2016). It has been observed that whole grain consumption has significant effects on medium‐ and long‐term parameters including reduction of fasting blood glucose and plasma insulin concentrations in diabetics (Aune et al. 2013; Xu et al. 2021; Zong et al. 2016). The EAT‐Lancet diet recommended a daily intake of 232 g of whole grains. In the present study, the type 2 diabetics consumed 53.23 (SD 78.63) g daily. The intake of whole grains lower than the recommended amount is probably related to the preference for white bread over whole wheat bread. It is also thought that whole grain choice may be influenced by educational status. The inability of individuals with low educational levels to identify whole grains has been identified as a barrier to consumption (Chea and Mobley 2019; Ruggiero et al. 2019). This may have been the case in the present study since most of the population had a primary school education.

It is known that added sugar and sugary drinks increase the risk of diabetes (Kawate et al. 1979). The EAT‐Lancet diet recommends a reference intake of 0–31 g of added sugar per day. In the present study, the participants consumed an average of 33.00 (SD 45.46) g of added sugar daily, which is close to the recommended intake. In comparison, in a study conducted with non‐diabetic individuals, mean sugar consumption of 56.67 (SD 32.26) g was reported, which is higher than the intake observed in our study (Stubbendorff et al. 2022). The high dietary adherence for daily added sugar in the present study may be attributed to the fact that the study population consisted of individuals with type 2 diabetes, who are likely to be more conscious of their sugar intake.

It has been demonstrated that nut consumption may play a role in the prevention and management of type 2 diabetes (Nishi et al. 2023). In the present study, nuts were the food group with the lowest dietary adherence, with a mean daily intake of 6.27 (SD 10.71) g, which is far below the recommendations of the EAT‐Lancet commission. This low adherence may be related to the high cost of nuts, concerns about weight gain, and issues such as dental problems or allergies that prevent some patients from consuming nuts. In previous studies, it was found that higher educational status is associated with higher nut consumption (Brown et al. 2014; Neale et al. 2020). The lower than recommended intake of nuts in our study may also be attributed to the low educational level of most of the study population.

Of the 385 type 2 diabetic volunteers who participated in the present study, 203 were female (52.7%) and 182 were male (47.2%). In previous studies conducted with healthy individuals, women were more adherent to the EAT‐Lancet diet than men were (Ibsen et al. 2022; Masip and Nielsen 2024; Zhang et al. 2023). In our study, the women with type 2 diabetes were more adherent to the diet than the men were based on their higher scores on the EAT‐Lancet Diet Index.

The International Diabetes Federation reports that the prevalence of diabetes by age is increasing. The aging of the world population has led to an increase in the prevalence of type 2 diabetics aged 65 years and older (Ogurtsova et al. 2022). According to the Türkiye Nutrition Health Surveys 2017 report, 26.4% of all type 2 diabetics were over 65 years of age (Satman et al. 2023). In the present study, 16.9% of the type 2 diabetics were over 65 years of age, and the majority (65.2%) were in the 46–65 age range. Ibsen et al. (2022) found that those who adopted the EAT‐Lancet diet at the highest level were younger. There are other studies in the literature showing that those who are most compliant with the EAT‐Lancet diet are older (Berthy et al. 2023; Stubbendorff et al. 2022). In our study, the men with type 2 diabetes who were most adherent to the EAT‐Lancet diet were older. In addition, a positive correlation was observed between age and adherence to the EAT‐Lancet diet.

Previous studies have shown that the rate of having a higher education level or university degree increases with increasing adherence to the EAT‐Lancet diet (Berthy et al. 2023; Ibsen et al. 2022; Stubbendorff et al. 2022; Zhang et al. 2023). In the present study, no statistically significant relationship was found between education level and diet adherence level.

The average energy intake taken as reference in the EAT‐Lancet diet is 2500 kcal/day. In the present study, the mean daily energy intake of the type 2 diabetics was 1429.61 (SD 417.29) kcal. The fact that the energy intake of individuals in our study was much lower than the reference energy intake may be attributed to several reasons, including the high mean age of the participants and the fact that they were not physically active. In addition, dietary 24‐h recall and food frequency questionnaire were used to collect food consumption data (Gunes et al. 2015; Pekcan 2008). These methods may cause memory bias or misrepresentation and may not reflect the actual consumption by the type 2 diabetics. Zhang et al. (2023) utilized the EAT‐Lancet Diet Index and observed that individuals with higher index scores were more likely to have lower total energy intake. In the present study, increased dietary adherence was associated with decreased daily energy intake.

In a study conducted in Sweden, those who showed the highest adherence to the EAT‐Lancet diet had lower daily energy, higher dietary fiber (g), lower fat (Energy %), higher carbohydrate (Energy %), and higher protein (Energy %) intake (Stubbendorff et al. 2022). In the present study, among all the type 2 diabetics who participated, those with the highest adherence to the EAT‐Lancet diet had a higher average daily intake of dietary fiber (g) compared to the others. In addition, the lowest adherent diabetics had a higher proportion of their daily energy intake derived from protein. Our findings are in line with those of the Swedish study for dietary fiber intake but differ for protein (Energy %) (Stubbendorff et al. 2022).

Several studies have found that the highest adopters of the EAT‐Lancet diet were more likely to have lower BMI and waist circumference (Ibsen et al. 2022; Masip and Nielsen 2024; Shojaei et al. 2024). A cohort study conducted in France revealed that those who showed the highest adherence to the EAT‐Lancet diet had a lower BMI (Berthy et al. 2023). In British adults, the highest dietary adherence was associated with a BMI of 1.4 kg/m^2^ lower (Knuppel et al. 2019). In a multicenter study across Latin America, no significant association was observed between the adherence to the EAT‐Lancet diet and prevalence of overweight or obesity (Vargas‐Quesada et al. 2025). In our study, an increase in adherence to the EAT‐Lancet diet was associated with a no significant increase in BMI and waist circumference and a decrease in waist/hip ratio.

No relationship was found between dietary adherence and blood glucose levels in the Iranian cohort (Shojaei et al. 2024). On the contrary, in this study, there was a statistically significant negative relationship between adherence to the EAT‐Lancet Diet and fasting blood glucose level (mg/dL). Although none of the individual predictors reached statistical significance in the regression model, the near‐significant association between diabetes duration and EAT‐Lancet Diet Index suggests a possible trend. The lack of significant associations with BMI, fasting glucose, and HbA1c indicates that adherence to the EAT‐Lancet Diet in this sample may be influenced by factors beyond clinical parameters, such as psychological, behavioral, or environmental determinants. Future research should incorporate a broader range of variables to better explain dietary adherence.

There are a number of limitations to the present study. Some of the data collected through the survey method were based on verbal statements. Individuals may have made false statements. In addition, the participants had a high mean age, and so the retrospective 24‐h dietary recall may have included incorrect recollections. Thus, their actual consumption might not be shown.

This study shows that high adherence to the EAT‐Lancet diet in individuals with type 2 diabetes is associated with improved clinical and nutritional indicators, including lower fasting blood glucose and HbA1c levels, higher dietary fiber intake and lower energy, fat and protein intake. These findings suggest that sustainable dietary patterns may not only support metabolic control, but also improve health status in individuals with type 2 diabetes. Besides improving individual health outcomes, adoption of the EAT‐Lancet diet may also contribute to environmental sustainability. Integrating the principles of sustainable nutrition into public health policies and clinical nutrition practice can provide significant benefits in diabetes management. Prospective studies with larger sample sizes are needed to better understand etiological relationships and to evaluate the long‐term effects of sustainable diets.

## Author Contributions

**Meltem Karabicak:** conceptualization (supporting), data curation (lead), formal analysis (equal), investigation (lead), methodology (equal), writing – original draft (lead). **Aysun Yuksel:** conceptualization (lead), data curation (supporting), formal analysis (equal), investigation (supporting), methodology (equal), writing – review and editing (lead).

## Disclosure

Declaration of generative AI and AI‐assisted technologies in the writing process: During the preparation of thiswork, the author(s) used ChatGPT (OpenAI GPT‐4) in order to improve language clarity and readability. After usingthis tool, the author(s) reviewed and edited the content as needed and take(s) full responsibility for the content ofthe publication.

## Ethics Statement

This study was conducted in accordance with the guidelines laid down in the Declaration of Helsinki, and all procedures involving human subjects were approved by the Hamidiye Scientific Research Ethics Committee (meeting number 2023/11, decision number 11/3 on 26.05.2023), University of Health Sciences, Istanbul, Türkiye. Written informed consent was obtained from all participants.

## Conflicts of Interest

The authors declare no conflicts of interest.

## Supporting information


**Table S1.** Energy and nutrient intake levels of individuals with type 2 diabetes (*n* = 385).
**Table S2.** Energy and nutrient intake levels of female with type 2 diabetes (*n* = 203).
**Table S3.** Energy and nutrient intake levels of male with type 2 diabetes (*n* = 182).

## Data Availability

The data that support the findings of this study are available on request from the corresponding author. The data are not publicly available due to privacy or ethical restrictions.
