# Supplementary material for: EAT‐Lancet Diet Adherence in Patients With Type 2 Diabetes: A Cross‐Sectional Study
Source: Food Sci Nutr. 2025 Jul 18;13(7):e70650. doi: 10.1002/fsn3.70650 (PMC12271971; doi:10.1002/fsn3.70650)
Supplement: Supplementary file 1 — Table S1. Energy and nutrient intake levels of individuals with type 2 diabetes (n = 385). Table S2. Energy and nutrient intake levels of female with type 2 diabetes (n = 203). Table S3. Energy and nutrient intake levels of male with type 2 diabetes (n = 182). [file FSN3-13-e70650-s001.docx]

**Supplementary Table-A.** Energy and Nutrient Intake Levels of Individuals with Type 2 Diabetes (n=385)

|  | **x̄** | **SD** | **Min.** | **Max.** | **Median** |
| --- | --- | --- | --- | --- | --- |
| **Energy (kcal)** | 1429,61 | 417,29 | 364,58 | 2858,38 | 1401 |
| **Protein (g)** | 59,5 | 18,48 | 10,59 | 118,21 | 58,83 |
| **Protein (%)** | 17,23 | 3,06 | 9,5 | 29 | 17 |
| **Oil (g)** | 63,38 | 19,19 | 20,71 | 145,34 | 60,88 |
| **Oil (%)** | 39,87 | 7,9 | 18 | 62 | 39,5 |
| **Carbohydrate (g)** | 151,71 | 62,01 | 24,76 | 378,23 | 141,33 |
| **Carbohydrate (%)** | 42,79 | 9,13 | 14,5 | 71 | 43 |
| **Fiber (g)** | 16,79 | 6,41 | 5,02 | 43,23 | 16,19 |
| **Alcohol (g)** | 0,36 | 2,91 | 0 | 52,35 | 0 |
| **Alcohol (%)** | 0,11 | 0,85 | 0 | 13,5 | 0 |
| **Polyunsaturated fatty acid** | 12,39 | 4,77 | 2,31 | 31,43 | 11,32 |
| **Cholesterol (mg)** | 315,22 | 170,49 | 40,64 | 1233,91 | 299,11 |
| **Vitamin A (meg)** | 1275,24 | 3291,22 | 124,16 | 37472,74 | 702,72 |
| **Carotene (mg)** | 1,85 | 1,45 | 0,23 | 15,67 | 1,46 |
| **Vitamin E (mg)** | 12,63 | 4,73 | 2,58 | 32,88 | 11,64 |
| **Thiamine (mg)** | 0,68 | 0,24 | 0,21 | 1,74 | 0,66 |
| **Riboflavin (mg)** | 1,28 | 0,48 | 0,24 | 4,8 | 1,23 |
| **Pyridoxine (mg)** | 0,9 | 0,32 | 0,21 | 3,73 | 0,86 |
| **Folate (mg)** | 254,75 | 88,24 | 72,14 | 797,48 | 244,48 |
| **Vitamin C (mg)** | 62,57 | 29,33 | 5,6 | 184,64 | 56,46 |
| **Sodium (mg)‡** | 2640,85 | 1635,64 | 355,47 | 28902,28 | 2471,42 |
| **Potassium (mg)** | 1708,23 | 467,59 | 547,37 | 3203,44 | 1693,36 |
| **Calcium (mg)** | 609,72 | 200 | 110,53 | 1907,4 | 602,49 |
| **Magnesium (mg)** | 198,49 | 65,83 | 71,64 | 445,67 | 188,01 |
| **Phosphorus (mg)** | 946,49 | 290,09 | 242,2 | 2382,47 | 934,72 |
| **Iron (mg)** | 7,05 | 2,51 | 2,23 | 18,27 | 6,61 |
| **Zinc (mg)** | 8,38 | 2,72 | 2,26 | 21,69 | 8,14 |
| **Soluble Fiber (g)** | 4,74 | 2 | 0,74 | 17,13 | 4,46 |
| **Insoluble Fiber (g)** | 10,96 | 4,52 | 1,99 | 31,76 | 10,22 |
| **Saturated Fatty Acids (g)** | 23,65 | 8,09 | 5,67 | 66,41 | 22,71 |
| **Monounsaturated Fat (g)** | 22,03 | 7,32 | 6,55 | 51,38 | 20,87 |
| **C18:2 Linoleic Acid (g)** | 10,62 | 4,47 | 1,41 | 27,63 | 9,46 |
| **C18:3 Linolenic Acid (g)** | 0,75 | 0,34 | 0,18 | 3,51 | 0,68 |
| **Glycemic Index** | 159,06 | 83,16 | 0 | 453,96 | 145,93 |

**Supplementary Table-B.** Energy and Nutrient Intake Levels of Female with Type 2 Diabetes (n=203)

|  | **x̄** | **SD** | **Min.** | **Max.** | **Median** |
| --- | --- | --- | --- | --- | --- |
| **Energy (kcal)** | 1278,02 | 333,47 | 364,58 | 2370,31 | 1260,08 |
| **Protein (g)** | 53,02 | 14,19 | 10,59 | 89,81 | 53,89 |
| **Protein (%)** | 17,29 | 3,04 | 9,5 | 27,5 | 17 |
| **Oil (g)** | 58,89 | 16 | 20,71 | 104,78 | 56,86 |
| **Oil (%)** | 41,32 | 7,64 | 25,5 | 62 | 40,5 |
| **Carbohydrate (g)** | 131,04 | 51,08 | 24,76 | 283,76 | 123,62 |
| **Carbohydrate (%)** | 41,4 | 8,98 | 14,5 | 63 | 41,5 |
| **Fiber (g)** | 14,93 | 5,29 | 5,02 | 43,23 | 14,64 |
| **Alcohol (g)** | 0,09 | 0,92 | 0 | 13,23 | 0 |
| **Alcohol (%)** | 0,02 | 0,38 | 0 | 5,5 | 0 |
| **Polyunsaturated fatty acid** | 11,21 | 4,36 | 2,31 | 26,74 | 10,24 |
| **Cholesterol (mg)** | 292,6 | 137,73 | 40,64 | 842,54 | 293,89 |
| **Vitamin A (meg)** | 1015,58 | 2310,3 | 228,96 | 24883,75 | 647,48 |
| **Carotene (mg)** | 1,87 | 1,59 | 0,23 | 15,67 | 1,46 |
| **Vitamin E (mg)** | 11,72 | 4,5 | 2,58 | 28,33 | 10,79 |
| **Thiamine (mg)** | 0,61 | 0,19 | 0,24 | 1,44 | 0,6 |
| **Riboflavin (mg)** | 1,17 | 0,34 | 0,27 | 2,78 | 1,175 |
| **Pyridoxine (mg)** | 0,81 | 0,22 | 0,33 | 1,45 | 0,79 |
| **Folate (mg)** | 233,68 | 68,87 | 83,67 | 447,35 | 230,49 |
| **Vitamin C (mg)** | 60,46 | 27,02 | 6,68 | 157,97 | 55,3 |
| **Sodium (mg)‡** | 2354,29 | 785,15 | 355,47 | 5262,3 | 2231,87 |
| **Potassium (mg)** | 1576,38 | 407,85 | 600,95 | 2765,47 | 1620,45 |
| **Calcium (mg)** | 579,76 | 178 | 110,53 | 1067,63 | 562,43 |
| **Magnesium (mg)** | 176,62 | 51,59 | 71,64 | 387,7 | 172,05 |
| **Phosphorus (mg)** | 863,02 | 223,02 | 246,26 | 1595,16 | 873,09 |
| **Iron (mg)** | 6,21 | 1,79 | 2,23 | 12,45 | 6,08 |
| **Zinc (mg)** | 7,48 | 1,98 | 2,26 | 13,07 | 7,5 |
| **Soluble Fiber (g)** | 4,14 | 1,58 | 1,34 | 12,51 | 4,1 |
| **Insoluble Fiber (g)** | 9,89 | 3,86 | 3,08 | 30,28 | 9,4 |
| **Saturated Fatty Acids (g)** | 22,14 | 6,59 | 5,67 | 45,7 | 21,3 |
| **Monounsaturated Fat (g)** | 20,68 | 6,33 | 6,55 | 42,25 | 19,78 |
| **C18:2 Linoleic Acid (g)** | 9,64 | 4,15 | 1,41 | 25,53 | 8,76 |
| **C18:3 Linolenic Acid (g)** | 0,67 | 0,25 | 0,18 | 2,45 | 0,64 |
| **Glycemic Index** | 132,7 | 67,26 | 0 | 378,25 | 122,31 |

**Supplementary Table-C.** Energy and Nutrient Intake Levels of Male with Type 2 Diabetes (n=182)

|  | **x̄** | **SD** | **Min.** | **Max.** | **Median** |
| --- | --- | --- | --- | --- | --- |
| **Energy (kcal)** | 1598,69 | 436,81 | 581,19 | 2858,38 | 1510,29 |
| **Protein (g)** | 66,74 | 20,01 | 16,01 | 118,21 | 64,89 |
| **Protein (%)** | 17,17 | 3,09 | 10,5 | 29 | 17 |
| **Oil (g)** | 68,39 | 21,16 | 23,02 | 145,34 | 65,55 |
| **Oil (%)** | 38,24 | 7,89 | 18 | 61 | 38,25 |
| **Carbohydrate (g)** | 174,75 | 65,07 | 54,36 | 378,23 | 162,38 |
| **Carbohydrate (%)** | 44,35 | 9,06 | 21 | 71 | 44,5 |
| **Fiber (g)** | 18,85 | 6,91 | 5,7 | 43,15 | 18,12 |
| **Alcohol (g)** | 0,67 | 4,11 | 0 | 52,35 | 0 |
| **Alcohol (%)** | 0,21 | 1,16 | 0 | 13,5 | 0 |
| **Polyunsaturated fatty acid** | 13,7 | 4,88 | 6,13 | 31,43 | 12,79 |
| **Cholesterol (mg)** | 340,46 | 198,19 | 45,69 | 1233,91 | 305,55 |
| **Vitamin A (meg)** | 1564,85 | 4106,6 | 124,16 | 37472,74 | 763,01 |
| **Carotene (mg)** | 1,84 | 1,27 | 0,23 | 7 | 1,45 |
| **Vitamin E (mg)** | 13,65 | 4,78 | 6,18 | 32,88 | 12,59 |
| **Thiamine (mg)** | 0,77 | 0,27 | 0,21 | 1,74 | 0,73 |
| **Riboflavin (mg)** | 1,4 | 0,57 | 0,24 | 4,8 | 1,33 |
| **Pyridoxine (mg)** | 1 | 0,38 | 0,21 | 3,73 | 0,94 |
| **Folate (mg)** | 278,26 | 100,87 | 72,14 | 797,48 | 264,73 |
| **Vitamin C (mg)** | 64,92 | 31,61 | 5,6 | 184,64 | 57,34 |
| **Sodium (mg)‡** | 2960,46 | 2189,3 | 622,74 | 28902,28 | 2698,95 |
| **Potassium (mg)** | 1855,28 | 486,79 | 547,37 | 3203,44 | 1813,2 |
| **Calcium (mg)** | 643,13 | 217,65 | 185,44 | 1907,4 | 637,09 |
| **Magnesium (mg)** | 222,89 | 71,34 | 75,88 | 445,67 | 214,09 |
| **Phosphorus (mg)** | 1039,6 | 326,34 | 242,2 | 2382,47 | 1011,22 |
| **Iron (mg)** | 7,99 | 2,84 | 2,3 | 18,27 | 7,49 |
| **Zinc (mg)** | 9,38 | 3,07 | 2,38 | 21,69 | 8,99 |
| **Soluble Fiber (g)** | 5,41 | 2,19 | 0,74 | 17,13 | 5,01 |
| **Insoluble Fiber (g)** | 12,15 | 4,9 | 1,99 | 31,76 | 11,47 |
| **Saturated Fatty Acids (g)** | 25,32 | 9,23 | 7,37 | 66,41 | 24,6 |
| **Monounsaturated Fat (g)** | 23,54 | 8,04 | 6,99 | 51,38 | 22,11 |
| **C18:2 Linoleic Acid (g)** | 11,72 | 4,56 | 3,9 | 27,63 | 10,67 |
| **C18:3 Linolenic Acid (g)** | 0,83 | 0,4 | 0,22 | 3,51 | 0,74 |
| **Glycemic Index** | 188,46 | 89,29 | 18,71 | 453,96 | 176,15 |
